# Infiltration of the spinal cord and peripheral nerves in multiple myeloma

**DOI:** 10.3389/fonc.2022.991246

**Published:** 2022-10-06

**Authors:** Xiaoyun Su, Xiangquan Kong, Xiangchuang Kong, Zuneng Lu, Chuansheng Zheng

**Affiliations:** ^1^ Department of Radiology, Union Hospital, Tongji Medical College, Huazhong University of Science and Technology, Wuhan, China; ^2^ Hubei Province Key Laboratory of Molecular Imaging, Wuhan, China; ^3^ Department of Neurology, Renming Hospital of Wuhan University, Wuhan, China

**Keywords:** multiple myeloma, spinal cord, peripheral nerves, infiltration, magnetic resonance neurography

## Abstract

**Background:**

Multiple myeloma (MM) is a hematological malignancy, and intramedullary spinal cord metastasis is extremely rare.

**Methods:**

Clinical and radiological data were collected from electronic medical records as well as a literature review of reported cases.

**Results:**

We report a rare case of IgA-LAM stage IIB MM with involvement of the spinal cord and peripheral nervous system. Laboratory studies showed elevated levels of serum β2-macroglobulin and cerebrospinal fluid protein. Electromyography revealed a demyelinating process with motor conduction blocks. On MRI, the lesions of MM bone marrow are characterized as a type of diffuse infiltration. MR neurography demonstrated an enhanced nodule in the thoracic segment with swelling of the cervicothoracic segments of the spinal cord. Moreover, swelling and hypertrophy of the entire nerve branchial, lumbosacral plexus, and cauda equina were detected, accompanied by myofascitis and denervated muscles. Ultimately, the condition of the patient deteriorated quickly and she died with a diagnosis of refractory MM.

**Conclusion:**

MRI not only has the advantage of displaying the primary involved site of the bone marrow but also facilitates detecting extramedullary hematopoietic MM, such as infiltrating sites of the central and/or peripheral nervous system.

## Introduction

Multiple myeloma (MM) is a hematological malignancy characterized by the abnormal proliferation of clonoplasma cells in the bone marrow (1). Patients with MM may present with a variety of neurological complications, including metabolic encephalopathy, spinal cord compression, spinal radiculopathy, and peripheral neuropathy (2–4). That was considered to be associated with the remote effects of monoclonal gamma-globulinopathy or amyloidosis (2, 5). Approximately 1%–2% of MM patients have been reported to metastasize to the central nervous system (CNS), either as leptomeningeal myelomatosis or as soft-tissue and intraparenchymal lesions (6, 7). However, intramedullary spinal cord metastases are extremely rare.

Here, we report a rare case of MM with involvement of the spinal cord, cauda equine, and peripheral nervous system (PNS). We describe an uncommon radiological manifestation, which on magnetic resonance imaging (MRI) was noted to be a small enhancing nodule in the thoracic segment of the spinal cord, accompanying a wide range of swelling cervicothoracic spinal cord with long T1 and T2 signals. Furthermore, swelling and hypertrophy of peripheral nerves were detected by MR neurography in the whole of the brachial and lumbosacral (LS) plexus, nerve roots and their branches with surrounding myofascitis and denervated muscles.

## Methods

The study was approved by the institutional ethics review board, and written informed consent was obtained from all patient patients. Clinical and radiological data were collected from electronic medical records as well as a literature review of reported cases.

## Results

### Case report

A 64-year-old woman initial presented with numbness of extremities about half a year, then aggravated to progressive quadriparesis, accompanying dysphagia, cough and hoarseness caused by nerve injury. The muscle forces were four grades in the lower extremities with hyperalgesia. Tendinous reflexes were dropped or disappeared, and the pathological sign was negative.

Laboratory studies showed hypoalbuminemia (albumin, 30.8 G/L) and an albumin globulin ratio decreased (A/G, 1.0). There was mild renal insufficiency with proteinuria (504 mg/24 h) and increased urea nitrogen (6.6 mmol/L). Hypocalcemia (0.75 mmol/L), hyperphosphatemia (110 mmol/L), and elevated levels of thyroid stimulating hormone, thyroid peroxidase antibody, and anti-TG were detected. Blood routine tests showed increased leucocytes (12.8 G/L), neutrophils (10.2 G/L), and erythrocyte sedimentation rate (34 mg/L), but without anemia. Then, lumbar puncture revealed a clear fluid with elevated protein at 0.81 g/L (normal range, 0.15–0.45 g/L) without plasma cells and glucose at 4.0 mmol/L with normal cell counts. Electromyography revealed a demyelinating process with motor conduction blocks on different nerves associated with markedly prolonged F-wave latency, particularly in the lower extremities.

As her symptoms, electrophysiological and cerebrospinal findings were compatible with a clinical diagnosis of suspected chronic inflammatory demyelinating polyneuropathy (CIDP) with acute exacerbation, a diagnostic treatment (i.e., intravenous immunoglobulin, IVIG) was carried out. However, she has responded poorly to IVIG treatment and progressively worsened at a local hospital. It means that more examinations were needed to obtain more evidence, such as spine MRI and immunoelectrophoresis. Then, an elevated level of vascular endothelial growth factor (VEGF) was detected in the serum. Moreover, magnetic resonance imaging (MRI) revealed abnormal findings that were hard to explain by CIDP.

### Imaging findings

**Figure 1:**
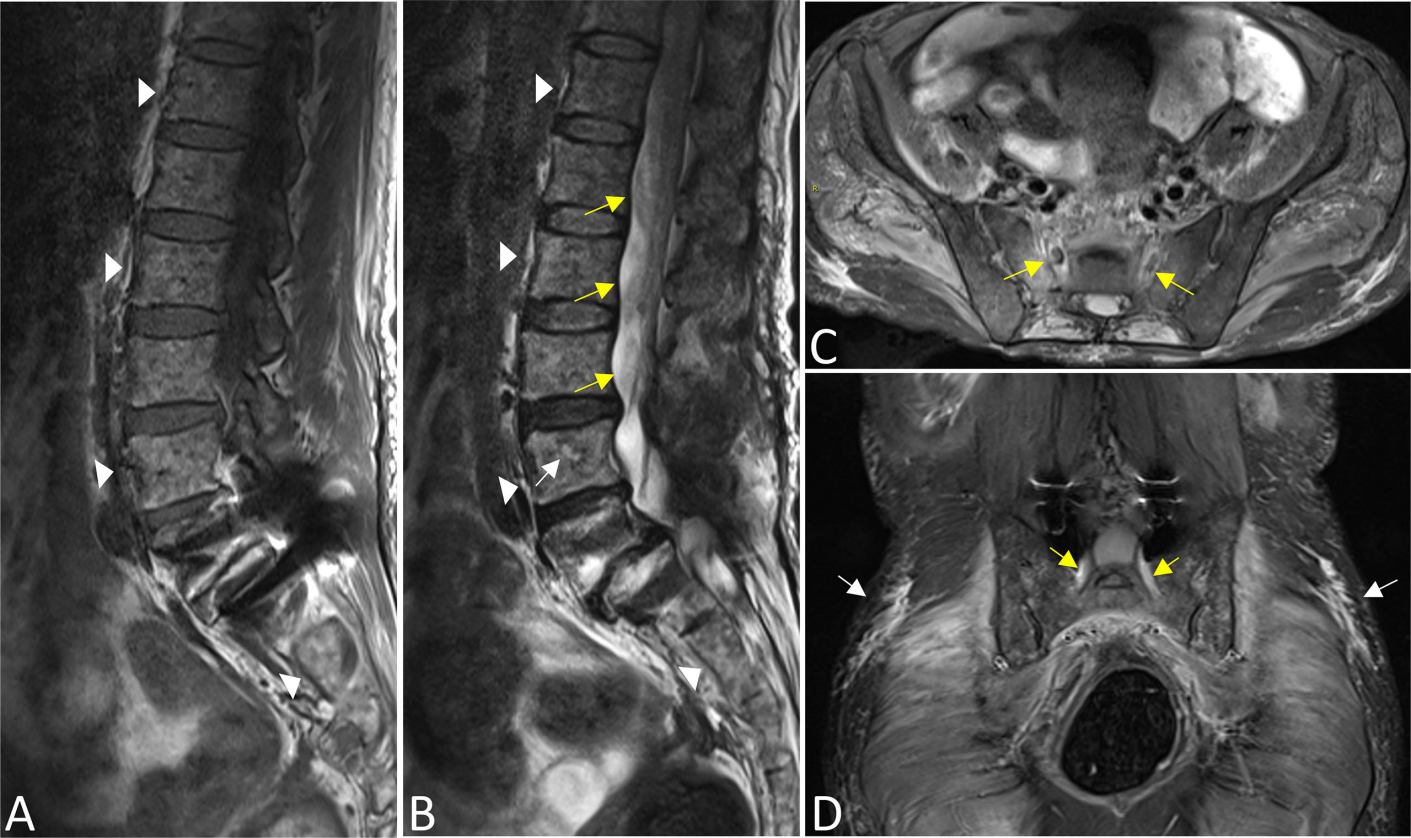
The bone marrow signals of the lumbosacral and caudal vertebrae were diffusely reduced on T1WI **(A)** and increased on T2WI images **(B)** that corresponds with the type of diffuse infiltration (triangles). A focal lesion with long T2 signal was seen in the body of the L4 vertebrae (white arrow). There was swelling of the cauda equina and terminus (yellow arrows in B). Swelling of bilateral S1 nerve roots was observed (yellow arrows in **C,D**), accompanied by a wide range of myofascitis and atrophy in denervated lumbar gluteal muscles on PDWI sequence (**C, D**, white arrows). In addition, the L5 vertebral body slipped forward I degree, and the surrounding tissues were affected by artifacts after internal fixation **(A, B, D)**.

**Figure 2:**
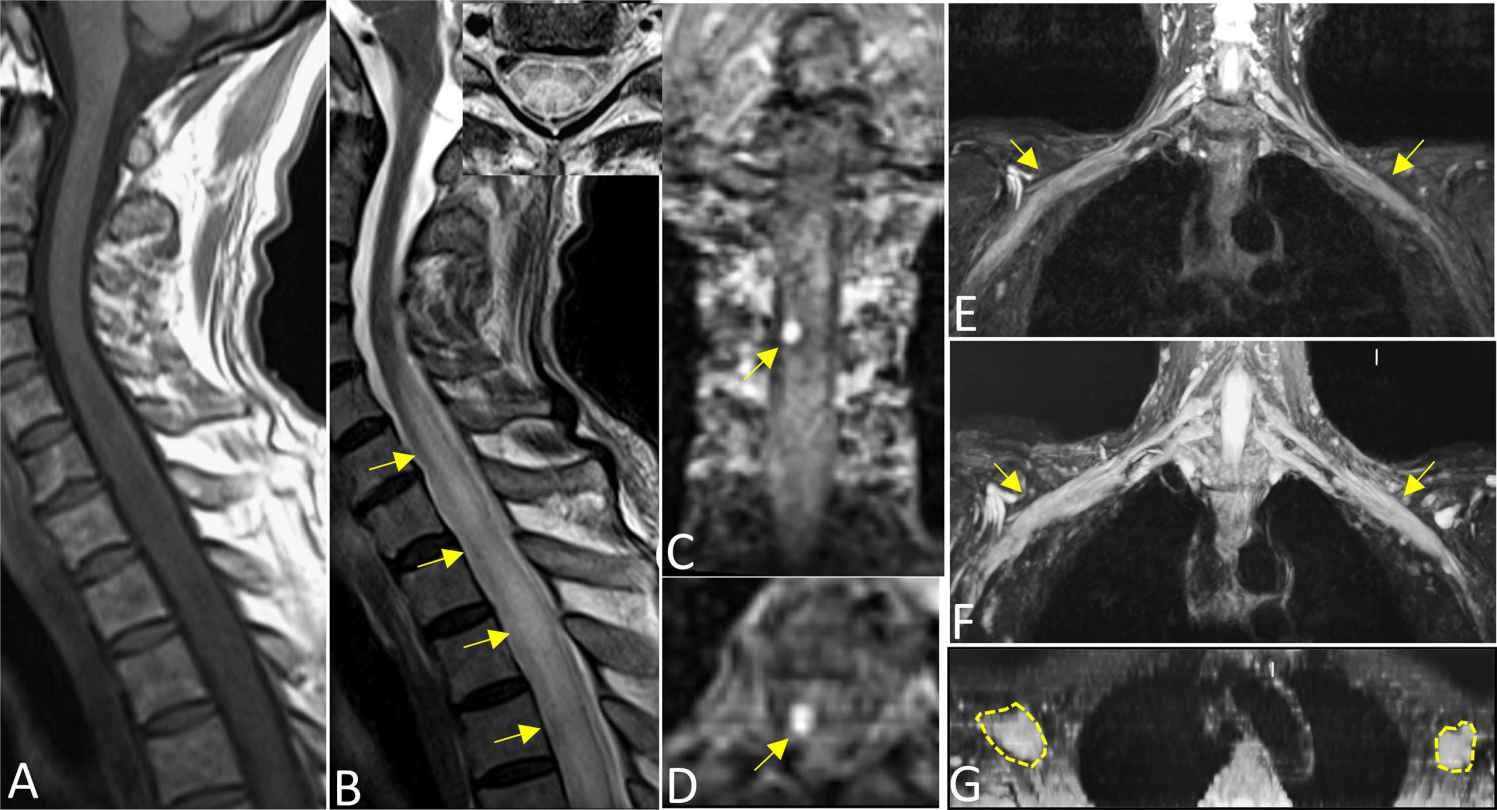
Sagittal T1WI **(A)** and T2WI images **(B)** revealed extensive swelling in the cervicothoracic segments of the spinal cord with long T1 and T2 signals (arrows). Axial magnified views **(B)** showed lesions both involving gray and white matter of the spinal cord and covering more than one-half of the cross-sectional area. Images **(C)** (coronal) and **(D)** (axial) showed an enhanced nodule with a diameter of about 7 mm in the thoracic segment of the spinal cord at the right Th3/4 intervertebral (arrows). Reconstructed magnetic resonance neurography revealed symmetrical remarkable swelling and hypertrophy of the brachial plexus, nerve roots, fascicles and their branches, with increased T2 signal intensity, especially in the fascicles (arrows in coronal **(E, F)**, circles outline in axial **G**).

On MRI, bone marrow signals of the lumbosacral and caudal vertebrae were diffusely reduced on T1-weighted images (T1WI) and increased on T2-weighted images (T2WI), which met the type of diffuse infiltration according to the published criteria (8) (**Figures 1A, B**). MRI showed markedly swollen cervicothoracic segments of the spinal cord with long T1 and T2 signals; the lesions were mainly located in thoracic segments and were relatively longer (**Figures 2A, B, D**). Notably, an enhanced nodule with a diameter of about 7 mm was detected in the thoracic segment of the spinal cord at the right Th3/4 intervertebral level after gadolinium administration (**Figures 2C, D**). In addition, there was swelling of the cauda equina and terminus (**Figure 1B**). Further, MR neurography revealed remarkable swelling and hypertrophy of the brachial plexus, nerve roots, fascicles, and their branches, with increased T2 signal intensity, especially in the fascicles (**Figures 2E–G**). Although affected by artifacts of the internal fixator, swelling of the LS plexus was observed, accompanied by a wide range of myofascitis and atrophy in denervated lumbar gluteal muscles on proton density weighted imaging (PDWI) sequence (**Figures 1C, D**).

Brain MR showed a few hypersignals of white matter in bilateral frontal lobes, without enhancement (**Supplementary Figures 1A, B**). Chest CT showed scattered inflammatory lesions of flake-shaped high-density on both lungs (**Supplementary Figure 1C**). 18F-FDG PET-CT images showed a mild distribution of radioactivity (maximum standardized uptake value, 3.0) on the lower thoracic vertebrae. No metastatic signs of malignant tumors were detected in the rest (**Supplementary Figure 2**).

The M-protein IgA-LAM was found in the serum by immunofixation. Serum β2-macroglobulin was increased to 4.0 mg/L. Further, a bone marrow biopsy of the iliac crest confirmed plasma cell infiltration and supported the definitive diagnosis of IgA-LAM stage IIB. Unfortunately, the condition of the patient deteriorated quickly by refusing further chemotherapy and immunotherapy treatment, and ultimately died with a diagnosis of refractory MM.

### Medical history

The woman had a history of hysterectomy for mature teratoma about 30 years ago and has undergone a lumbar spondylolisthesis fixation one year ago.

## Discussion

Involvement of the CNS by plasma cells is rare, either as leptomeningeal myelomatosis or intraparenchymal and soft-tissue lesions, accounting for only 1%–2% of MM patients (4, 6, 7). However, intramedullary spinal cord metastases are extremely rare. Although extramedullary plasmacytoma either forming soft tissue or directly invading the spinal canal or nerve roots in patients with MM has been previously reported in several studies (9–12). We report a rare case of MM with uncommon radiographic findings, characterized by diffuse involvement of the cervicothoracic segments of the spinal cord, cauda equine, and PNS.

Our case is unique because it presents as a small-enhanced nodule in the thoracic segment of the spinal cord, with extensive swelling and edema of the cervicothoracic segments, which has not been previously reported. Moreover, the cauda equina is characterized by diffuse swelling. To our knowledge, only two cases of diffuse infiltration in the cauda equina have been reported previously (9, 12). However, 18F-FDG PET-CT only found mildly focal increased metabolism in the vertebral body and failed to detect any intramedullary lesion, which might be attributed to its insensitivity to the nervous tissue. At present, several theories have been proposed for how MM infiltrates the CNS. It is suggested that extramedullary plasmacytoma of the CNS is due to hematogenous spread through the arachnoid vein (13). Another hypothesis holds that continuous spread arising directly from adjacent osteolytic lesions is supported by neuroimaging evidence (14). But so far, there is no general agreement and exact molecular pathogenic mechanisms have yet to be investigated.

Peripheral neuropathy is one of the most important and common complications in patients with MM. Peripheral neuropathy can be caused by MM itself through the effects of the monoclonal protein or in the form of radiculopathy due to direct compression or especially by certain chemical treatments, while the pathological mechanism is not well understood (15, 16). Neuropathies are associated with MM in 3%–5% of the patients, and most patients have peripheral nerve-related symptoms, signs, or have electrophysiological characteristics (17, 18). The patients presented with progressive symmetrical sensory-motor polyneuropathy, simulating Guillain–Barré syndrome or CIDP, characterized by reduced nerve conduction velocity and conduction block. In such cases, electrophysiological tests could be misleading in the initial diagnosis. The cause of polyneuropathy in this case was related to complications of treatments that were not considered since the patient only received a course of IVIG treatment and did not receive any radiotherapy or chemotherapy.

To our knowledge, MR neurography, as an advanced peripheral neuroimaging technique, has not been used or reported in patients with MM. In this case, MR neurography revealed symmetrical swelling and hypertrophy with increased signal intensity without enhancement in the plexus, nerve roots, fasciculus and its branches, which might be similar to CIDP to some extent. However, a swollen nerve was prominently located at the fasciculus in this case, whereas nerve roots are more common in CIDP (19). In addition, note the effusion and swell of the perineurium, accompanied by a wide range of myofascitis and atrophy in denervated muscles, even more remarkable than CIDP, possibly related to the cachexia of plasma cells. These might be helpful to differentiate from the CIDP and warrant further studies with a larger sample size. The findings suggest that MR neurography may be useful in detecting extramedullary infiltration in MM, especially for either the CNS or PNS, which has predominant use in evaluating the entire nerve tracts. Moreover, previous autopsy or biopsy studies have demonstrated that malignant infiltrations of the peripheral nerves by plasma cells in MM patients with symptomatic neuropathy (20, 21). Unfortunately, we failed to obtain confirmatory pathological results because peripheral nerve biopsy was refused by this patient.

In this case, the lesions of MM bone marrow are characterized as the type of diffuse infiltration according to the published criteria (8). It is well-known that MRI has the advantage of detecting the primary site of involvement in MM, i.e., the bone marrow, and further facilitates the staging of the disease (11, 22), and our result was in line with the literature. Synthetically considered among the results of clinical, radiological, histopathological, and laboratory findings, the monistic approach of the hematologist attributes the abnormalities in the spinal cord and peripheral nerves mostly to MM infiltration. The condition of the patient deteriorated quickly by refusing further chemotherapy and immunotherapy treatments, and ultimately died with a diagnosis of refractory MM. That indicates a worse prognosis in patients with extramedullary infiltration of the nervous system.

There are several differential diagnoses of this patient with MM intramedullary infiltration that should be considered. Firstly, it needs to be differentiated from peripheral neuropathy such as CIDP and Guillain–Barré syndrome, as these would mimic symptoms and laboratory findings. Secondly, polyneuropathy, organomegaly, endocrinopathy, monoclonal plasma cell disorder, and skin changes (POEMS) syndrome serves as a multisystem disease associated with underlying plasma cell neoplasm, which rarely involves the spinal cord. Finally, it is necessary to differentiate between myelitis and neoplasms of the spinal cord. However, the former usually does not show nodular enhancement on MRI, and the latter does not often cause spinal cord swelling of long segments or involve extensive bone lesions and peripheral nervous system.

In conclusion, we reported a rare case of MM with an uncommon radiographic presentation, suggesting that MRI not only has the advantage of displaying the primary involved site of the bone marrow but also facilitates detecting infiltrating sites of the CNS and/or PNS in MM. Moreover, extramedullary hematopoietic MM infiltrating the CNS and PNS often portends a poor prognosis.

## Data availability statement

The raw data supporting the conclusions of this article will be made available by the authors, without undue reservation.

## Ethics statement

The present prospective study was approved by the ethics committee of Tongji Medical College, Huazhong University of Science and Technology (No. IORG0003571). The patients/participants provided their written informed consent to participate in this study.

## Author contributions

Conceived and designed: CZ, XS, and ZL. Analyzed the data: XS and XQK. Manuscript preparation: XS and CZ. Contributed reagents/materials: ZL and XCK. All authors contributed to the article and approved the submitted version.

## Funding

This work was supported by the National Natural Science Foundation of China (Grant Nos. 81470076 and 81701653). 

## Conflict of interest

The authors declare that the research was conducted in the absence of any commercial or financial relationships that could be construed as a potential conflict of interest.

## Publisher’s note

All claims expressed in this article are solely those of the authors and do not necessarily represent those of their affiliated organizations, or those of the publisher, the editors and the reviewers. Any product that may be evaluated in this article, or claim that may be made by its manufacturer, is not guaranteed or endorsed by the publisher.
