# Supplementary material for: Infiltration of the spinal cord and peripheral nerves in multiple myeloma
Source: Front Oncol. 2022 Oct 6;12:991246. doi: 10.3389/fonc.2022.991246 (PMC9584647; doi:10.3389/fonc.2022.991246)
Supplement: Supplementary file 1 [file DataSheet_1.pdf]

SUPPLEMENTARY FIGURE

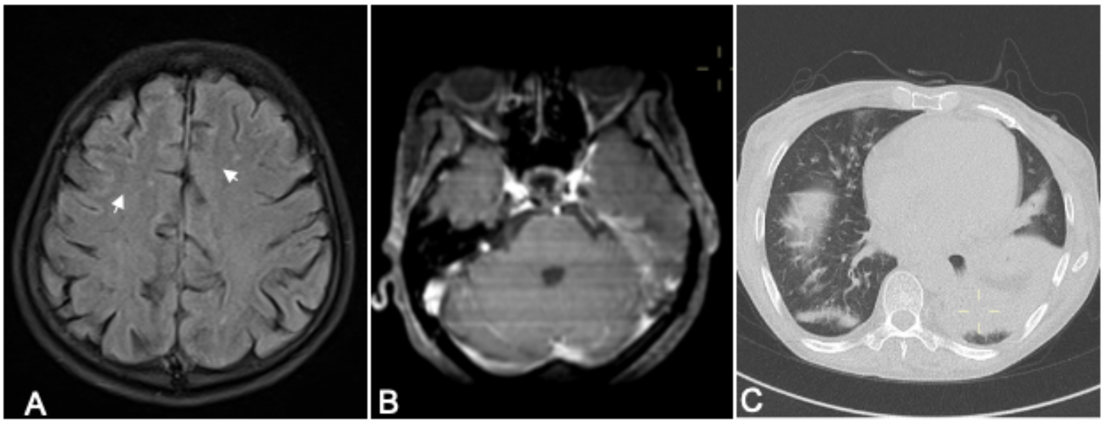

Supplementary figure 1. Image A (FLAIR) showed a few hypersignals of white matter in bilateral frontal lobes (arrows). No abnormal enhancement was observed after Gd administration (B). Chest CT (C) showed scattered inflammatory lesions of the flake-shaped high-density on both lungs.

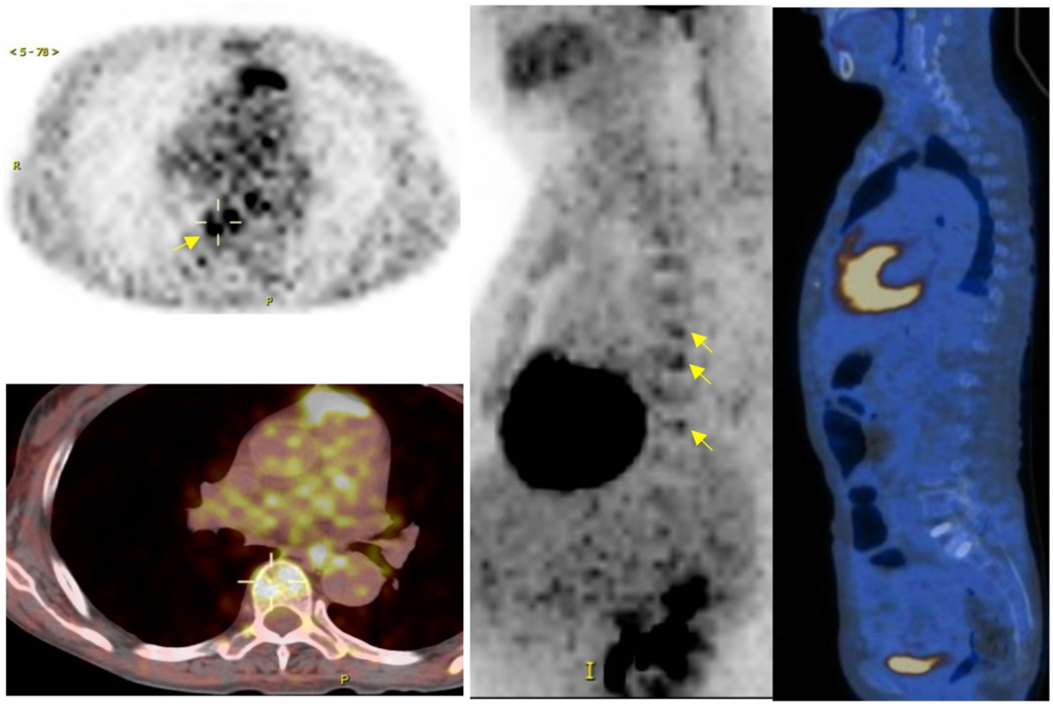

Supplementary figure 2. 18F-FDG PET images showed mildly distribution of radioactivity (maximum standardized uptake value, 3.0) on lower thoracic vertebrae. No metastatic signs of malignant tumor were detected in the rest.

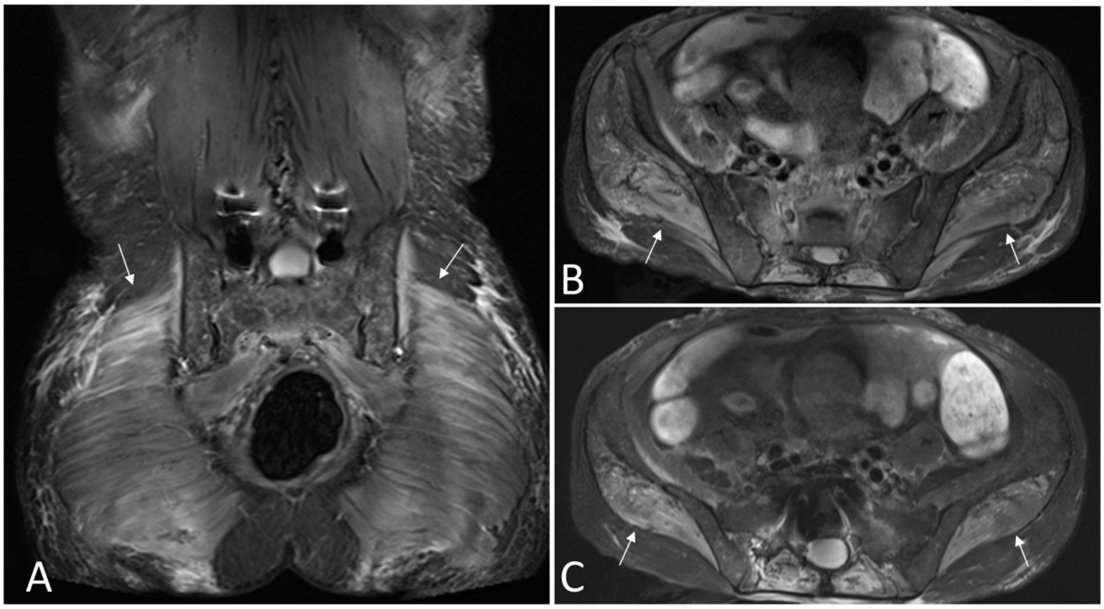

Supplementary figure 3. A wide range of myofascitis and atrophy in denervated lumbar gluteal muscles on the PDWI sequence (white arrows).
